# Lacto-ovo-vegetarian diet is inversely associated with the osteosarcopenia in older adults

**DOI:** 10.1186/s12877-024-04959-6

**Published:** 2024-04-11

**Authors:** Runnian Huang, Junwei Hu, Yi Li, Sijia Yang, Xin Li, Tianbo Hou, Zibo Ning, Chunhua Ma, Xiaoyue Yuan, Zheng Wang, Tiantian Zhang, Difei Wang

**Affiliations:** 1 Department of Gerontology and Geriatrics, Shengjing Hospital of China Medical University, 110004 Shenyang, Liaoning China; 2 Department of Health Statistics, School of Public Health, China Medical University, 110122 Shenyang, Liaoning China; 3 Department of Geriatric Cardiology, The First Hospital of China Medical University, 110001 Shenyang, Liaoning China

**Keywords:** Dietary patterns, Osteosarcopenia, Older adults, Weighted quantile sum regression, Principal component analysis

## Abstract

**Objective:**

Osteosarcopenia adversely affects the quality of life and physical health of older adults. We sought to explore the association between dietary patterns and osteosarcopenia in community-dwelling older adults.

**Methods:**

This is a cross-sectional study from Northeast China, in which, we included older community adults aged 60 and above. Through face-to-face interviews, we collected dietary information from participants using a food frequency questionnaire. Subsequently, principal component analysis (PCA) was used to obtain the dietary patterns of the participants. Through physical examination, we obtained the participants’ information on osteosarcopenia, which was defined by the coexist of osteopenia and sarcopenia. We analysed the association between dietary patterns and dietary compositions with ostesarcopenia.

**Results:**

In this study, a total of 9429 participants were included, and the prevalence of osteosarcopenia in community-dwelling older adults was 6.2%. PCA identified three main dietary patterns, and the lacto-ovo-vegetarian dietary pattern was inversely associated with osteosarcopenia. Compared to the lowest lacto-ovo-vegetarian quartile (Q1), the Q4 group (OR = 0.64, 95% CI:0.49–0.83) was inversely associated with osteosarcopenia. Through the weighted quantile sum regression model, we also found that the overall effect of the lacto-ovo-vegetarian dietary components was inversely associated with osteosarcopenia (OR = 0.58, 95% CI: 0.37–0.92); the largest contributors were vegetables, fresh milk, eggs, and dairy products.

**Conclusion:**

Overall, we found that a lacto-ovo-vegetarian dietary pattern, particularly the consumption of vegetables, fresh milk, eggs, and dairy products, was inversely associated with osteosarcopenia in older adults. And this might provide new insights for the prevention and treatment of osteosarcopenia.

**Supplementary Information:**

The online version contains supplementary material available at 10.1186/s12877-024-04959-6.

## Introduction

In the human body, the bones and muscles interact to form the skeletal muscular system, which shapes the human body, maintains body posture, allows simple and advanced movements, protects important organs, and facilitates metabolic storage. However, with age, a decline in BMD (bone mineral density), SMI (skeletal muscle mass index), and muscle strength leads to an increased risk of osteopenia and sarcopenia. These common geriatric diseases with shared risk factors contribute to the onset of aging and associated adverse health outcomes, such as frailty syndrome [1, 2].

Recently, an emerging and unique geriatric syndrome, osteosarcopenia, has been investigated. It is characterized by low BMD and sarcopenia [3, 4] and it significantly increases the risk of fractures, falls, and mortality [5]. Compared to osteopenia/osteoporosis or sarcopenia, relatively limited epidemiological data on osteosarcopenia are available [2]. Current evidence indicates that the worldwide prevalence of osteosarcopenia is between 4.7–40% [6], with variability among study populations [3, 7]. However, as the world population ages, the prevalence of osteosarcopenia will inevitably increase, leading to frailty, fractures, falls, hospitalization, and deaths.

There are currently no drugs for treating osteosarcopenia [3, 7, 8]; however, nutrition and diet have been shown to affect bone and muscle health. Poor eating habits and nutritional status in older adults, particularly frail older adults, are important causes of osteopenia and sarcopenia. A high-quality diet is essential for the development and maintenance of bone and muscle health [3, 7, 9, 10]. Vitamin D and calcium simultaneously promote bone and muscle health, while dietary proteins are important in maintaining bone and muscle integrity [11]. Studies on single nutrients or food can only provide limited information on the complexity of human dietary behaviour. Conversely, studying dietary patterns allows the simultaneous evaluation of the intake and interrelationships of multiple nutrients and foods; this is more useful in the formulation of dietary intervention strategies [12, 13]. At present, research methods on dietary patterns are predominantly divided into two categories [14]: literature or guidelines-based dietary indices, such as dietary diversity scores, Mediterranean dietary patterns, and healthy eating indices and specific dietary patterns obtained from data aggregation and analysis, such as principal component analysis (PCA) and cluster analysis.

Recently, some researchers have applied analytical methods to high-dimensional data, such as LASSO regression [15] and weighted quantile sum (WQS) regression models [16], to dietary research. Dietary patterns constructed from PCA do not consider outcome variables. Therefore, the factor loading of dietary components only represents the contribution that makes up the dietary pattern, and the relationship between dietary components and outcomes cannot be derived. The WQS regression model not only estimates the overall effect of environmental exposure, but also assesses the contribution of each individual component. We believe that combining the PCA and WQS regression model can compensate for their respective shortcomings.

Previous studies have explored the association between dietary patterns and osteopenia or sarcopenia [17, 18], which reported that “Healthy” and “Milk/dairy” patterns were associated with a decreased risk of low BMD, whereas the “Meat/Western” patterns showed a significant positive association with low BMD, and mediterranean diet was positively associated with muscle function in older adults. Besides, a previous study reported the relationship between lacto-ovo-vegetarian diet and sarcopenic-obesity [19], however, to our knowledge, no studies have yet investigated the relationship between dietary patterns and osteosarcopenia. Therefore, in the present study, we aimed to obtain the dietary patterns of community-dwelling older adults in Northeast China, and explore the association between different dietary patterns and osteosarcopenia. In addition, we also aimed to explore the relationship between dietary components and osteosarcopenia under specific dietary pattern.

## Materials and methods

### Study design and setting

This cross-sectional study was conducted in Northeast China. Data were collected from 12,519 community-dwelling adults in 2019 and 2021. Both from March to October in 2019 and 2021 years, we conducted face-to-face interviews and physical examinations for community-dwelling older adults. In our study, community is the fundamental unit of resident life and urban governance, emphasizing the regional community. A community has approximately 30,000 to 50,000 people, with relatively complete public facilities and venues. Residents living in the same community often have similar lifestyles and socio-economic status. And the communities were randomly selected from Shenyang city, Liaoning Province. The research team included two of professors of geriatrics, several attending physicians in geriatrics, dozens of medical graduate students, and general practitioners from community health service centres.

### Participants

**Fig. 1:**
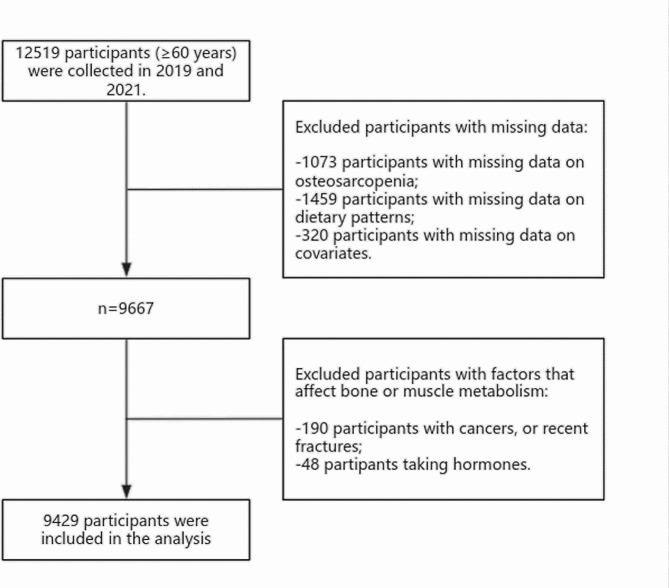
The flow chart of the study

Participants were recruited from each community through community health service centres, which have health records for every resident. Medical staff from community health service centres recruit eligible participants by telephone and schedule face-to-face surveys and physical examinations. Participants were older adults in the community in Shenyang, Liaoning Province, aged ≥ 60 years. Subsequently, we excluded participants with missing data and those who may have factors affecting the bone and muscle metabolism (Fig. 1).

### Procedures

Before the investigation, all investigators participated in the training and passed the assessment. Through face-to-face questionnaires, we collected the demographic information, lifestyle information, history of chronic diseases and medication history of participants. And through physical examinations, we measured the participants’ height, weight, body composition, hand grip (HG), gait speed (GS), and BMD.

### Dietary assessment

In the present study, we collected participants’ food intake information over the past year using a validated [19, 20] self-reported FFQ. The survey included 22 food groups that represent Chinese eating habits and nutritional needs and recorded the frequency of intake for each food group; nine categories of frequency were included, ranging from never consumed to three or more times daily.

### Definition of osteosarcopenia

In the present study, osteosarcopenia was defined as osteopenia combined with sarcopenia [3]. Given the conditions of our research group, we measured participants’ heel BMD T values [21, 22] using an ultrasonic densitometer (BMTECH, OsteoPro UBD2002A, South Korea) and defined osteopenia as a T value ≤-1, however peripheral bone densitometry was often used to evaluate osteoporosis or osteopenia [23], and peripheral BMD results were highly predictive of fracture risk [24]. Sarcopenia was diagnosed as a combination of the following: low SMI, low muscle strength, and/or low physical performance [25]. Consistent with another previous publication by our research group [19], as we measured body skeletal muscle mass (SMM) using a bioelectrical impedance analysis using an HBF-701 body composition analyser (Omron, Kyoto, Japan) rather than appendage skeletal muscle (SMI was calculated as SMM divided by height squared), the cut-off value was selected as the mean of body SMI in healthy adults minus two standard deviations [26, 27]. Low SMI was defined as < 5.78 kg/m^2^ in men and < 4.58 kg/m^2^ in women. Low HG was used to represent muscle strength, defined as < 28 kg in men and < 18 kg in women [25]. Low GS was defined as < 1.0 m/s regardless of sex (GS = 6/TUG time *1.62) [28], and was used to represent low physical performance.

### Covariates

Participants wore light clothing and no shoes, so that their weight and height could be accurately measured (accurate to 0.01 cm and 0.01 kg). Body mass index (BMI) was calculated as weight (kg) divided by height in meters squared (m^2^). The remaining covariates were obtained by professional clinical investigators through face-to-face participant interviews using standardised questionnaires. The questionnaire included gender (male/female), age, ethnicity (Han/minority), education level (illiterate, primary school, middle school, high school and above), income level (0-1000yuan, 1000-3000yuan, 3000 + yuan), self-reported history of smoking, drinking, and chronic diseases. Exercising levels were assessed using the International Physical Activity Questionnaire and divided into rarely (< 1 times/week), sometimes (1–3 times/week), often (≥ 4 times/week).

### Statistical analysis

To generate dietary patterns, we first calculated the daily consumption frequency of 22 food groups in the FFQ [20]. Subsequently, the frequencies were entered into PCA and varimax (orthogonal) rotation was applied for greater interpretability. The Kaiser-Meyer-Olkin (KMO) test of sampling adequacy (> 0.6) and Bartlett’s sphericity test (*P* < 0.05) were used to judge the applicability of the data to PCA [19, 29]. The number of principal components (dietary patterns) retained was based on the eigenvalues (> 1.0), first inflection point of the scree plots, and interpretability of the components [29]. Dietary pattern scores (higher scores indicate higher compliance) were calculated by summing the standardised food consumption frequencies weighted by the corresponding factor loading. Food items with absolute values of factor loadings greater than 0.3 were considered as the primary contributors to the dietary patterns. We subsequently grouped dietary patterns according to quartiles of their scores for subsequent analysis.

The Kolmogorov-Smirnov test revealed that the continuous variables were not normally distributed. Descriptive data were illustrated using median and interquartile range for continuous data and frequencies as percentages for categorical data. The Kruskal-Wallis test and chi-square test or Fisher’s exact test was used to compare continuous and categorical variables between groups. We estimated the odds ratio (OR) and 95% CI using logistic regression models. The median scores of the dietary pattern quartile groups were used as continuous variables to test the p for trend. In addition, we used the Wald test to assess whether the observed relationship was linear or nonlinear and constructed spline plots to describe the dose-response relationship between the dietary pattern score and the OR (95%CI) of osteosarcopenia. Subgroup analyses were designed to assess the potential impact of sociodemographic and lifestyle factors by adding interaction terms to the fully adjusted model. These regression models were as follows: crude model included single dietary pattern; model 1 was adjusted for other dietary patterns, gender and age; model 2 was additionally controlled for ethnicity, education, income, smoke, drink, exercise, and BMI; besides, in model 3, we additionally controlled for chronic diseases including hypertension, diabetes, hyperlipidemia, coronary heart disease, stroke and chronic obstructive pulmonary disease, and controlled for investigated year to eliminate potential impacts of time factor.

Subsequently, we used the WQS regression model [16, 30] to assess the magnitude of the effects of various dietary components in a particular dietary pattern on the overall effect. In simple terms, the WQS regression model constructs a weighted index in a supervised manner to assess the overall effect of environmental exposure and the contribution of each component using the following formula:

$$ \text{W}\text{Q}\text{S}=\sum _{\text{i}=1}^{\text{c}}{{\upomega }}_{\text{i}}{\text{q}}_{\text{i}}$$

$${\rm{g}}\,\left( {\rm{\upmu }} \right)\,{\rm{ = }}\,{{\rm{\upbeta }}_{\rm{0}}}\,{\rm{ + }}\,{{\rm{\upbeta }}_{\rm{1}}}\,{\rm{*}}\,{\rm{WQS}}\,{\rm{ + }}\,{{\rm{z}}^{\rm{'}}}\,{\rm{\upvarphi }}$$

Here, $\text{c}$ indicates the number of dietary components, ${{\upomega }}_{\text{i}}$ represents the weight (ranging from 0 to 1), and $\sum {\upomega }=1$; ${\text{q}}_{\text{i}}$ represents the quartiles of each dietary component. $\text{g}\left({\upmu }\right)$ is a logit link function, which is binary here (with or without osteosarcopenia);${ {\upbeta }}_{0}$ is the intercept; ${{\upbeta }}_{1}$ is the regression coefficient of the WQS index; ${\text{z}}^{{\prime }}$ and ${\rm{\upvarphi }}$ represent the matrix of covariates and the coefficients of the covariates, respectively. The weight size of each dietary component indicates its contribution to the overall effect of the WQS index. The data were randomly split into 40% as a training set and 60% as a validation set. Subsequently, the receiver operating characteristic curve (ROC) and area under the curve (AUC) were used to validate the degree of WQS regression model fit.

All statistical tests and charts were performed using R (version 4.3.1), and statistical significance was set at *P* < 0.05.

## Results

Before analysis, we excluded 1,073 participants with missing data on the definition of osteosarcopenia, 1,459 participants lacking the complete food frequency questionnaire (FFQ), 320 participants with incomplete demographic information, 190 participants with cancers or recent fractures, and 48 participants taking hormones. Finally, a total of 9,429 participants were included in the analysis (Fig. 1).

### Dietary patterns of older adults in the community

**Fig. 2:**
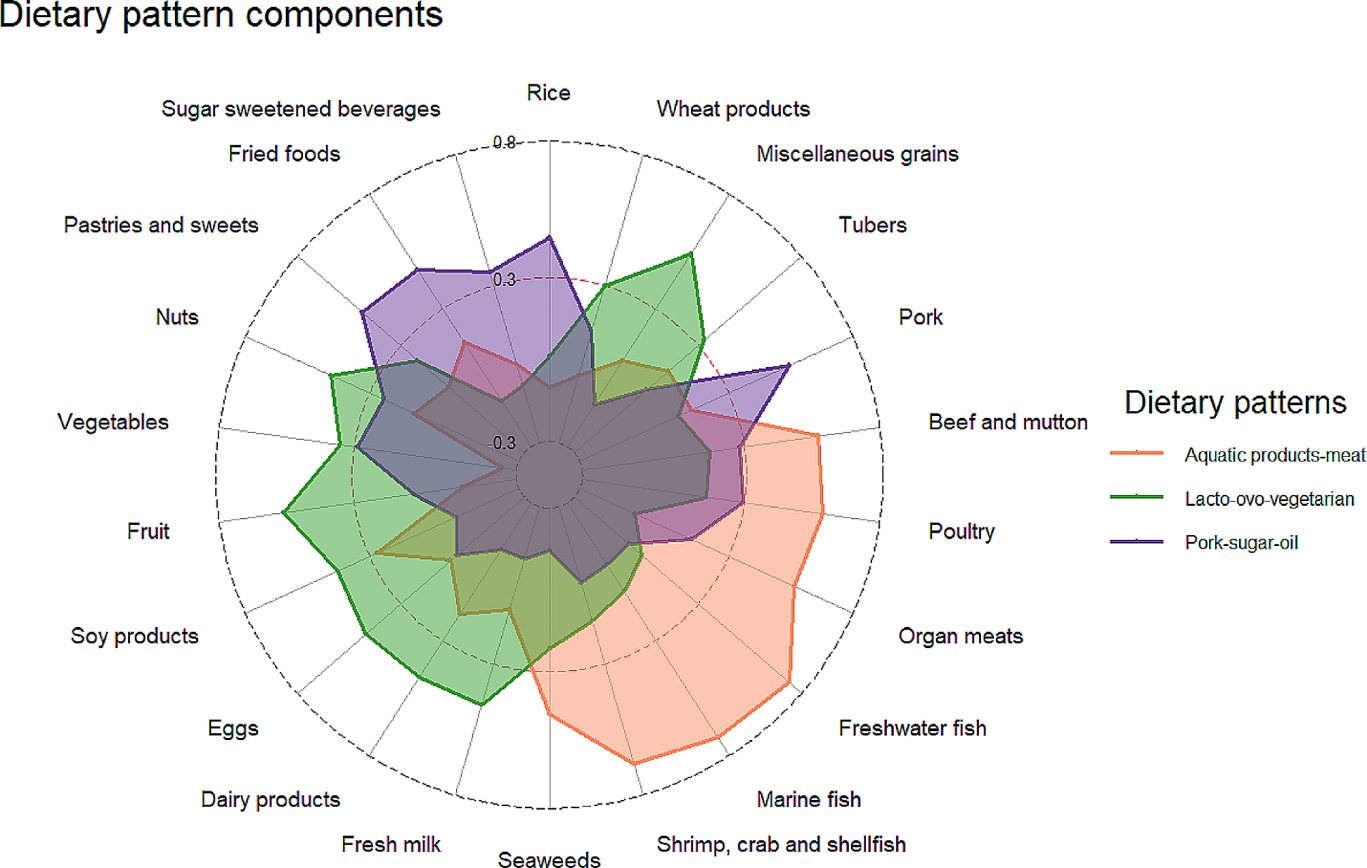
The composition of the three primary dietary patterns

According to the KMO test (0.77) and Bartlett’s sphericity test (*P* < 0.001), 22 food groups in FFQ were eligible for PCA. Based on the eigenvalues, scree plots (Supplementary Fig. 1), and interpretability, three main dietary patterns, which accounted for 30.4% of the total variation, were extracted from the PCA. These were aquatic products-meat dietary pattern (the first three largest factor loadings were freshwater fish, marine fish and shrimp, crab, and shellfish), lacto-ovo-vegetarian dietary pattern (fruit, miscellaneous grains, and eggs), and pork-sugar-oil dietary pattern (pork, pastries and sweets, and fried foods). These dietary patterns explained 13.8%, 9.8%, and 6.8% of the variations, respectively. Their compositions as well as the factor loadings were shown in Fig. 2 and Supplementary Table 1.

### Baseline characteristics of the participants

Of the total of 9429 participants in this study, 593 (6.2%) had osteosarcopenia, 5929 (62.8%) were women, and the median age was 68 years old. The statistical descriptions grouped by the scores of dietary patterns were shown in Supplementary Tables 2–4.

Table 1 presents the baseline characteristics, grouped by osteosarcopenia. Compared to participants without osteosarcopenia, those with osteosarcopenia more likely to be older, male, with lower or higher income level, smoker, drinker, and with lower BMI. There were no between-group differences for the remaining factors (*P* > 0.05).

**Table 1:** Baseline characteristics of the participants grouped by osteosarcopenia

	No	Yes	p
	*N* = 8836	*N* = 593	
Age	68.0[65.0;72.0]	70.0[66.0;75.0]	< 0.001
Gender			< 0.001
Male	3074(34.8%)	426(71.8%)	
Female	5762(65.2%)	167(28.2%)	
Ethnicity			0.127
Han	8376(94.8%)	553(93.3%)	
Minority	460(5.21%)	40(6.75%)	
Education level			0.204
Illiterate	205(2.32%)	17(2.87%)	
Primary school	1084(12.3%)	82(13.8%)	
Middle school	4477(50.7%)	275(46.4%)	
High school and above	3070(34.7%)	219(36.9%)	
Income level			0.003
0-1000 yuan	483(5.47%)	36(6.07%)	
1000–3000 yuan	4125(46.7%)	234(39.5%)	
3000 + yuan	4228(47.8%)	323(54.5%)	
Exercising level			0.268
Rarely	1460(16.5%)	83(14.0%)	
Sometimes	532(6.02%)	38(6.41%)	
Often	6844(77.5%)	472(79.6%)	
Smoke			< 0.001
Yes	1770(20.0%)	248(41.8%)	
No	7066(80.0%)	345(58.2%)	
Drink			< 0.001
Yes	1581(17.9%)	207(34.9%)	
No	7255(82.1%)	386(65.1%)	
BMI group			< 0.001
< 18.5 kg/m2	83(0.94%)	66(11.1%)	
< 24 kg/m2	3081(34.9%)	368(62.1%)	
< 28 kg/m2	4009(45.4%)	147(24.8%)	
≥ 28 kg/m2	1663(18.8%)	12(2.02%)	

### Associations between dietary patterns and osteosarcopenia

We used logistic regression models to assess the associations between dietary patterns and osteosarcopenia (Table 2). Within the aquatic products-meat and pork-sugar-oil dietary pattern, no association was observed between these two dietary patterns and osteosarcopenia. However, we found that the group with higher scores of the lacto-ovo-vegetarian dietary pattern was inversely associated with osteosarcopenia. In crude model, the Q2 (OR = 0.74, 95%CI: 0.59–0.92), Q3 (OR = 0.68, 95%CI: 0.54–0.86) and Q4 (OR = 0.67, 95%CI: 0.53–0.84) group of lacto-ovo-vegetarian diet were inversely associated with osteosarcopenia (p for trend < 0.001). And after adjusting for covariates, these associations were still significant. In the fully adjusted model, compared to the group of lowest score of lacto-ovo-vegetarian (Q1), Q2 (OR = 0.71, 95%CI: 0.55–0.91), Q3 (OR = 0.67, 95%CI: 0.52–0.87) and Q4 (OR = 0.64, 95%CI: 0.49–0.83) group remained inversely associated with osteosarcopenia (p for trend < 0.001).

**Table 2:** The associations between dietary patterns and osteosarcopenia

		Q1	Q2		Q3		Q4		p for trend
			OR	95%CI	OR	95%CI	OR	95%CI	
Crude model	Aquatic products-meat	ref	0.98	(0.77,1.24)	1.03	(0.81,1.30)	0.97	(0.77,1.23)	0.875
	Lacto-ovo-vegetarian	ref	0.74	(0.59,0.92)	0.68	(0.54,0.86)	0.67	(0.53,0.84)	< 0.001
	Pork-sugar-oil	ref	1.11	(0.87,1.41)	1.10	(0.87,1.40)	1.14	(0.90,1.45)	0.325
Model 1	Aquatic products-meat	ref	0.89	(0.70,1.14)	0.92	(0.72,1.17)	0.80	(0.62,1.02)	0.081
	Lacto-ovo-vegetarian	ref	0.73	(0.58,0.92)	0.67	(0.53,0.85)	0.64	(0.50,0.81)	< 0.001
	Pork-sugar-oil	ref	1.00	(0.78,1.28)	0.95	(0.74,1.22)	0.94	(0.73,1.20)	0.561
Model 2	Aquatic products-meat	ref	0.88	(0.68,1.13)	0.93	(0.72,1.21)	0.77	(0.59,1.00)	0.070
	Lacto-ovo-vegetarian	ref	0.71	(0.56,0.91)	0.68	(0.53,0.87)	0.61	(0.47,0.79)	< 0.001
	Pork-sugar-oil	ref	1.00	(0.77,1.30)	0.98	(0.78,1.28)	0.95	(0.73,1.24)	0.676
Model 3	Aquatic products-meat	ref	0.91	(0.70,1.17)	0.99	(0.76,1.28)	0.89	(0.68,1.16)	0.513
	Lacto-ovo-vegetarian	ref	0.71	(0.55,0.91)	0.67	(0.52,0.87)	0.64	(0.49,0.83)	< 0.001
	Pork-sugar-oil	ref	0.95	(0.73,1.24)	0.91	(0.69,1.19)	0.85	(0.65,1.11)	0.215

*Note* Crude model only included single dietary pattern; Model 1 adjusted for gender, age, and other dietary patterns; Model 2 was additionally controlled for ethnicity, education, income, smoke, drink, exercise, and BMI; Model 3 additionally controlled for chronic diseases including hypertension, diabetes, hyperlipidemia, coronary heart disease, stroke and chronic obstructive pulmonary disease, and controlled for investigated year to eliminate potential impacts of time factor

**Fig. 3:**
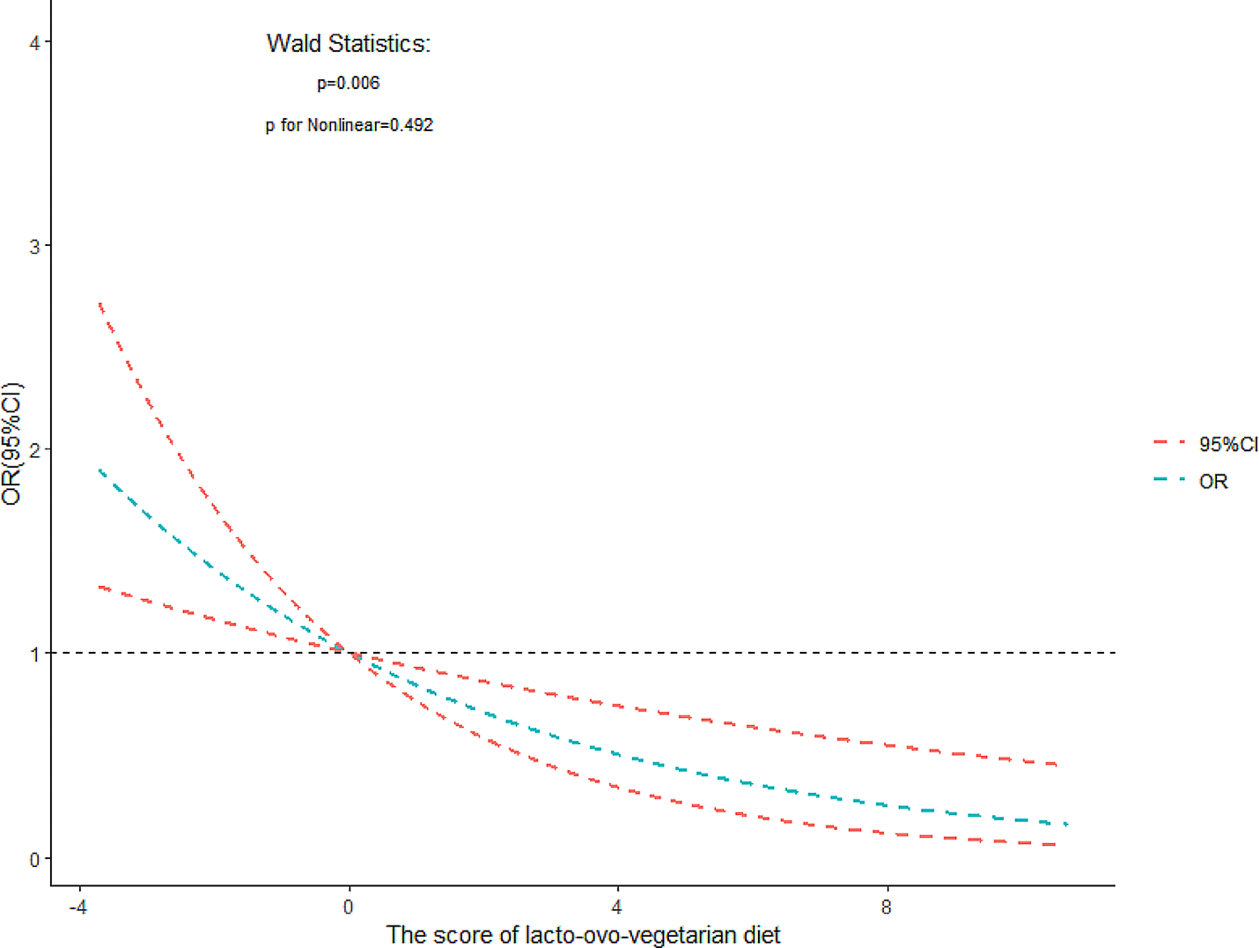
The relationship between the score of lacto-ovo-vegetarian diet and the OR of osteosarcopenia

Given this result, subsequent analyses were only focused on lacto-ovo-vegetarian dietary pattern. Through the Wald test, we found an approximate linear relationship between the lacto-ovo-vegetarian dietary pattern score and the OR of osteosarcopenia (Fig. 3). As the score of lacto-ovo-vegetarian diet was increasing, the OR of osteosarcopenia was decreasing (*p* = 0.006, p for Nonlinear = 0.492).

We also performed stratified analyses and observed associations of quartiles groups of lacto-ovo-vegetarian diet with osteosarcopenia in subgroups defined by gender, age, education level, income level, exercising level, smoke, drink, and BMI group. We merged some subgroups during analysis to avoid a small sample size for individual subgroups (Supplementary Figs. 2–9). However, we did not observe a significant interaction between the lacto-ovo-vegetarian diet and the main selected covariates with osteosarcopenia (all p for interaction > 0.05).

### Associations between the lacto-ovo-vegetarian diet and the components of osteosarcopenia

Subsequently, we used logistic regression models to explore the associations between the lacto-ovo-vegetarian dietary pattern and components of osteosarcopenia (Table 3). After adjusting for covariates, the results were found to be significant. In model 3, the Q4 group of the lacto-ovo-vegetarian dietary pattern was inversely associated with low BMD (OR = 0.88, 95% CI: 0.77–0.99, p for trend = 0.047), low SMI (OR = 0.70, 95% CI: 0.56–0.87, p for trend = 0.002), low GS (OR = 0.77, 95% CI: 0.68–0.87, p for trend < 0.001), and low HG (OR = 0.73, 95% CI: 0.65–0.83, p for trend < 0.001).

**Table 3:** Associations between the lacto-ovo-vegetarian diet and the components of osteosarcopenia

	Q1	Q2		Q3		Q4		p for trend
		OR	95%CI	OR	95%CI	OR	95%CI	
Low BMD								
Crude model	ref	0.98	(0.87,1.11)	0.95	(0.85,1.08)	0.89	(0.79,1.01)	0.054
Model 1	ref	0.97	(0.86,1.10)	0.92	(0.82,1.04)	0.85	(0.75,0.96)	0.010
Model 2	ref	0.99	(0.87,1.12)	0.96	(0.85,1.09)	0.88	(0.78,1.00)	0.066
Model 3	ref	0.99	(0.88,1.12)	0.96	(0.84,1.09)	0.88	(0.77,0.99)	0.047
Low SMI								
Crude model	ref	0.77	(0.64,0.93)	0.75	(0.62,0.90)	0.73	(0.60,0.88)	0.001
Model 1	ref	0.77	(0.64,0.94)	0.76	(0.62,0.92)	0.73	(0.60 0.89)	0.002
Model 2	ref	0.74	(0.60,0.91)	0.75	(0.60,0.92)	0.69	(0.55,0.85)	0.001
Model 3	ref	0.74	(0.59,0.91)	0.74	(0.60,0.92)	0.70	(0.56,0.87)	0.002
Low GS								
Crude model	ref	0.91	(0.81,1.02)	0.80	(0.71,0.90)	0.71	(0.63,0.80)	< 0.001
Model 1	ref	0.88	(0.78,0.99)	0.75	(0.67,0.85)	0.64	(0.57,0.73)	< 0.001
Model 2	ref	0.94	(0.83,1.05)	0.83	(0.74,0.94)	0.73	(0.64,0.83)	< 0.001
Model 3	ref	0.94	(0.83,1.06)	0.83	(0.73,0.94)	0.77	(0.68,0.87)	< 0.001
Low HG								
Crude model	ref	0.94	(0.84,1.05)	0.83	(0.74,0.93)	0.77	(0.69,0.86)	< 0.001
Model 1	ref	0.92	(0.82,1.03)	0.78	(0.69,0.87)	0.71	(0.63,0.80)	< 0.001
Model 2	ref	0.94	(0.83,1.05)	0.81	(0.72,0.91)	0.74	(0.66,0.84)	< 0.001
Model 3	ref	0.94	(0.84,1.05)	0.81	(0.72,0.92)	0.73	(0.65,0.83)	< 0.001

*Abbreviations* BMD: bone mineral density; SMI: skeletal muscle mass index; GS: gait speed; HG: hand grip

### WQS regression model of the dietary components

**Fig. 4:**
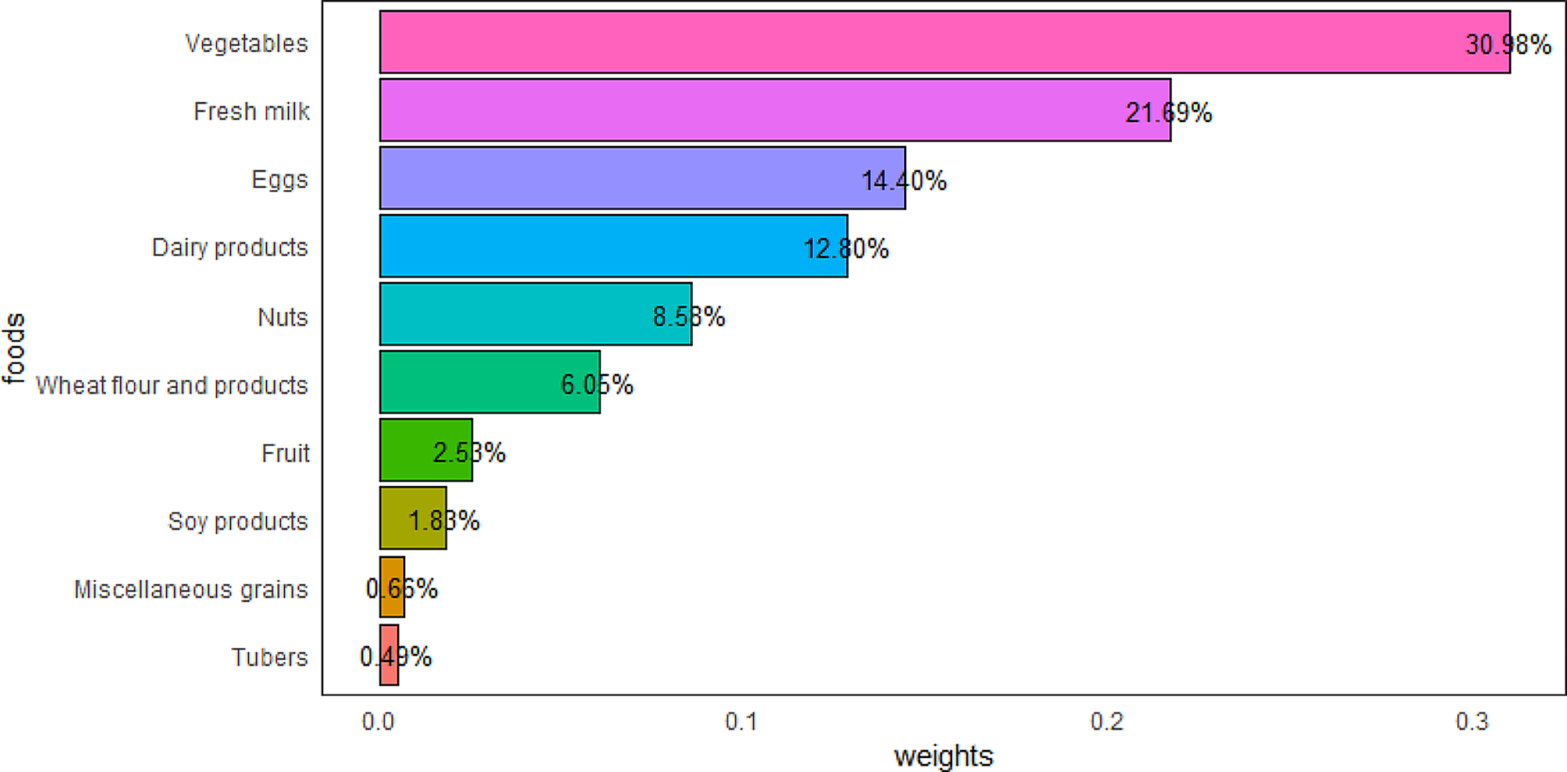
The weights of the dietary components in the weighted quantile sum regression model

**Fig. 5:**
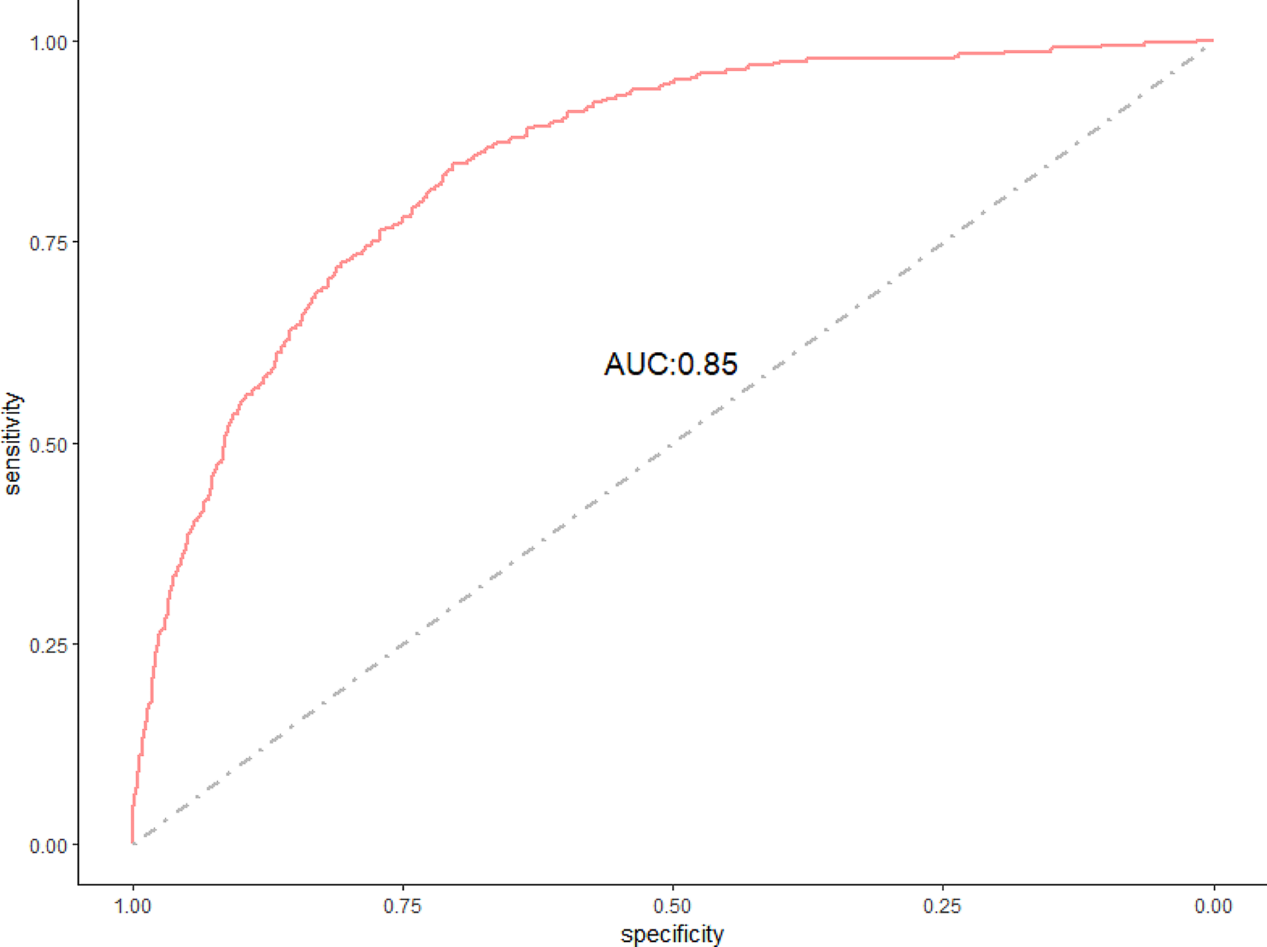
Receiver operating characteristic curve of the weighted quantile sum regression model

The WQS regression model was used to evaluate the health contributions of various dietary components of the lacto-ovo-vegetarian dietary pattern. After fully adjusting for the covariates, we developed a WQS regression model on 60% of the validation set and found that the WQS index (the overall effect of these dietary components) was inversely associated with osteosarcopenia (OR = 0.58, 95% CI: 0.37–0.92). Dietary component weight in the WQS regression model represented contribution to the overall effect; vegetables (30.98%), fresh milk (21.69%), eggs (14.40%), and dairy products (12.80%) were the most important dietary components (Fig. 4). The area under the ROC curve (AUC) was 0.85 (Fig. 5).

## Discussion

In this cross-sectional study of community older adults in Northeast China we identified three primary dietary patterns using PCA as follows: an aquatic products-meat dietary pattern, a lacto-ovo-vegetarian dietary pattern, and a pork-sugar-oil dietary pattern, accounting for 30.4% of the dietary variations in the population. Compared to a previous study [19] on the relationship between dietary patterns and sarcopenic-obesity, only slight differences were found in the components of these three dietary patterns (**Fig. 2**), which suggests they are representative of the dietary habits of community-dwelling elder individuals in northeast China. Besides, we extended this perspective by observing the relationship between lacto-ovo-vegetarian and osteosarcopenia. In subsequent analyses, we found that groups with higher scores on the lacto-ovo-vegetarian dietary pattern were inversely associated with of osteosarcopenia and its components (low BMD, low SMI, low HG, and low GS).

In the present study, the prevalence of osteosarcopenia in older adults was found to be 6.2%. This is within the range of 4.7–40% reported in the literature [6]. These differences in prevalence may be due to inconsistent diagnostic strategies as well as to variations in dietary habits and lifestyles in different populations.As shown in Table 2, the higher quartile group of lacto-ovo-vegetarian diet was inversely associated with osteosarcopenia. A previous study of older adults in South Korea [31] found that people with osteosarcopenia had a lower dietary protein intake. The primary components of the lacto-ovo-vegetarian dietary pattern are good sources of high-quality protein, which not only improves bone composition and mass, but can also promote bone formation by affecting insulin-like growth factor I secretion and activity [10]. Amino acids from dietary protein stimulate muscle synthesis and balance muscle loss in older adults [32]. This partly explains the protective effect of the lacto-ovo-vegetarian dietary pattern on osteosarcopenia.

We propose that the associations of lacto-ovo-vegetarian dietary pattern and osteosarcopenia may be explained by protecting against bone loss, loss of muscle mass, muscle strength, and physical function. As shown in Table 3, the Q4 group of the lacto-ovo-vegetarian dietary pattern was inversely associated with low BMD, indicating that this diet may protect against bone loss and promote bone health. This result is similar to the findings of a previous study of postmenopausal women in Iran [33], which reported that adherence to the dietary approaches to stop hypertension dietary pattern (DASH) was inversely associated with the risk of osteoporosis. The DASH diet involves a higher intake of fruit, vegetables, nuts and legumes, low-fat dairy products, and whole grains as well as a lower intake of sodium, sweets, and red or processed meats. The benefits of a lacto-ovo-vegetarian diet on bone health may also be partly due to the high concentrations of calcium and minerals within dietary components such as nuts, dairy and soy products, and some vegetables and fruit. Calcium is required for bone mineral matrix formation and to maintain bone strength, whilst potassium and magnesium participate in calcium metabolism and promote positive calcium balance. Magnesium is also involved in the activation of vitamin D, helping to maintain bone structure [10]. In addition, vegetables, fruit, and soy products provide the human body with vitamin K, which is involved in bone matrix formation, and vitamin C, an antioxidant that improves bone health by inhibiting osteoclast activity and increasing osteoblast differentiation(10). In addition, some fruits and vegetables are rich in phytoestrogens, such as lignans and isoflavones, which can reduce BMD loss due to oestrogen deficiency [34].

Conversely, we also found that the group with higher lacto-ovo-vegetarian dietary pattern scores was inversely associated with low SMI, low HG, and low GS (Table 3). A previous study [35] found that adherence to a healthy Nordic diet, characterised by high consumption of Nordic fruit, vegetables, whole-grain products, fish, and rapeseed oil, and low consumption of red meat and alcohol, could prevent muscle weakening in older women, although this was not observed in men. In addition, other studies have found that a higher intake of fruit and vegetables, milk, and dairy products was associated with a reduced risk of a lower HG [36–39], low GS [36, 39] and low SMI [38, 40]. Unlike the healthy Nordic diet, the lacto-ovo-vegetarian dietary pattern does not contain fish, but is rich in fresh milk, dairy products, miscellaneous grains, eggs, nuts, and soy products, which can also provide the body with abundant dietary protein. Fresh milk and eggs are the most bioavailable high-quality protein sources, while eggs are low in calories and high in leucine: a strong stimulator of muscle protein synthesis, which can directly promote muscle protein synthesis in older adults, increase skeletal muscle anabolic activity, and thus benefit muscle health [32, 41]. In addition to providing high-quality protein, the lacto-ovo-vegetarian dietary pattern provides the body with antioxidants such as vitamin C, vitamin E, and polyphenols. These compounds can reduce skeletal muscle inflammation and damage caused by high oxidative stress, thereby promoting and maintaining muscle mass, muscle strength, and physical performance [42–44]. In addition, a large intake of fruit and vegetables can neutralise the acidic degradation of muscle proteins and promote the synthesis of new muscle protein [45].

Interestingly, we did not observe an association between the protein-rich aquatic products-meat dietary pattern and osteosarcopenia. This may be related to several factors: First, red and processed meats are considered pro-inflammatory foods, implying that they increase inflammatory marker levels, which may contribute to the development of sarcopenia [46], and the decrease in BMD [47]. Second, people with higher scores on this dietary pattern were more likely to smoke and drink alcohol than the lowest-scoring group (Supplementary Tabel 2), which is detrimental to both bone and muscle health [2]. Finally, high meat consumption produces a lot of acid, leading to an acidic environment that promotes muscle protein breakdown and inhibits muscle protein synthesis [45]. However, our findings do not completely exclude such an association; further research is needed to explore the association between this diet and osteosarcopenia.

Further, in the WQS regression model, we found that the overall effect of the dietary components that make up the lacto-ovo-vegetarian dietary pattern was inversely associated with osteosarcopenia (OR = 0.58, 95% CI: 0.37–0.92). This further demonstrated a potential protective effect of the lacto-ovo-vegetarian diet against osteosarcopenia. Subsequently, we found that vegetables, fresh milk, eggs, and dairy products were the most influential components of this effect (**Fig. 4**). After evaluating the WQS regression model using the ROC curve, we concluded that this model had a good fit (**Fig. 5**).

This study has some limitations. First, due to the cross-sectional nature of the study, it was not possible to determine the causal relationship between the lacto-ovo-vegetarian dietary pattern and osteosarcopenia; more prospective studies are needed to confirm this relationship in the future. Second, FFQ only measured the frequency of food intake and not the amount, therefore, it was not possible to accurately calculate food intake. Third, this study used an ultrasound densitometer to measure the heel bones of participants rather than a dual-energy X-ray (DXA) densitometer to measure the BMD of the lumbar spine or hip bone. Although the densitometer is not the gold standard for diagnosing osteopenia or osteoporosis, it is nevertheless a rapid, safe, relatively inexpensive test that is suitable for the assessment of large samples; results are highly correlated with fracture risk [24], lumbar spine measurement and spine DXA results [21]. We believe that it has greater health and economic implications in population-based screening.

Despite these limitations, this study also has several strengths. First, to our knowledge, this is the first study to explore the association between dietary patterns and osteosarcopenia in older community-dwelling adults and lays the groundwork for future research. Second, compared with single nutrients and foods, the dietary patterns analysed in this study are more applicable to the formulation of dietary strategies. Third, the interviewers in this study were professionally trained and evaluated, and intensive training was conducted regularly. Fourth, the WQS regression model is traditionally used for environmental effect assessment and is less used for diet-related studies. In the present study, we combined PCA and WQS regression model to analyse both the association between dietary components and dietary patterns, and the relationship between specific components and health outcomes.

## Conclusions

In summary, higher lacto-ovo-vegetarian dietary pattern scores were inversely associated with osteosarcopenia, especially the intake of vegetables, fresh milk, eggs, and dairy products, which provided a new perspective for the prevention and treatment of osteosacropnia in older adults, as well as a new perspective for preventing geriatric syndromes from a daily dietary strategy. However, more prospective studies should be conducted in the future to validate the potential protective effects of a lacto-ovo-vegetarian diet against osteosarcopenia.

### Electronic supplementary material

Below is the link to the electronic supplementary material.

Supplementary Material 1

Supplementary Material 2

Supplementary Material 3

Supplementary Material 4

Supplementary Material 5

Supplementary Material 6

Supplementary Material 7

Supplementary Material 8

Supplementary Material 9

Supplementary Material 10

Supplementary Material 11

Supplementary Material 12

Supplementary Material 13

## Data Availability

The datasets used and/or analysed during the current study are available from the corresponding author on reasonable request.

## Abbreviations

PCA: principal component analysis
WQS: weighted quantile sum
BMD: bone mineral density
SMI: skeletal muscle mass index
FFQ: food frequency questionnaire
HG: hand grip
GS: gait speed
BMI: body mass index
OR: odds ratio
CI: confidence interval
ROC: receiver operating characteristic curve
AUC: area under the curve
