# Supplementary figures and images for: Lacto-ovo-vegetarian diet is inversely associated with the osteosarcopenia in older adults
Source: BMC Geriatr. 2024 Apr 11;24:332. doi: 10.1186/s12877-024-04959-6 (PMC11007993; doi:10.1186/s12877-024-04959-6)

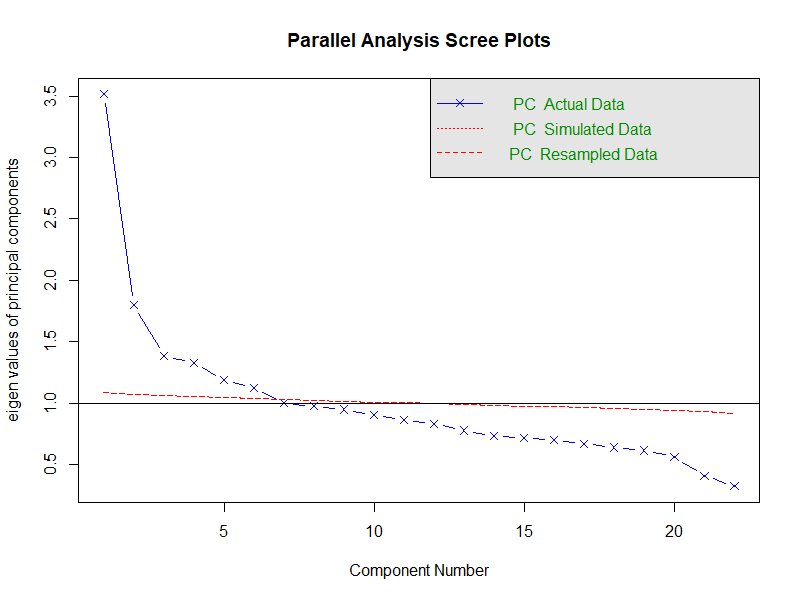

Supplement: Supplementary file 1 — Supplementary Material 1 [file 12877_2024_4959_MOESM1_ESM.tiff]

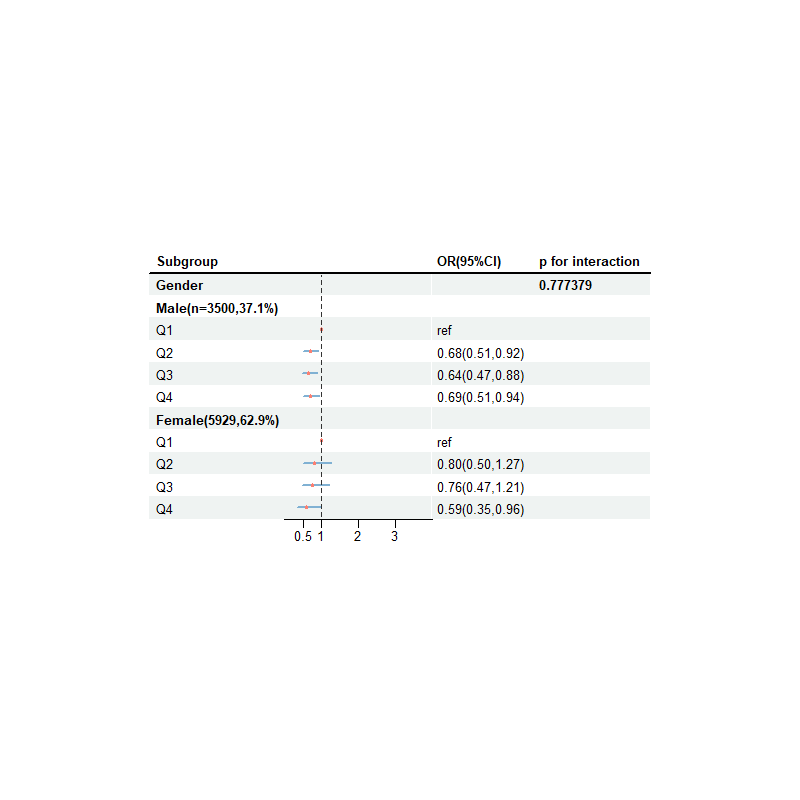

Supplement: Supplementary file 2 — Supplementary Material 2 [file 12877_2024_4959_MOESM2_ESM.tiff]

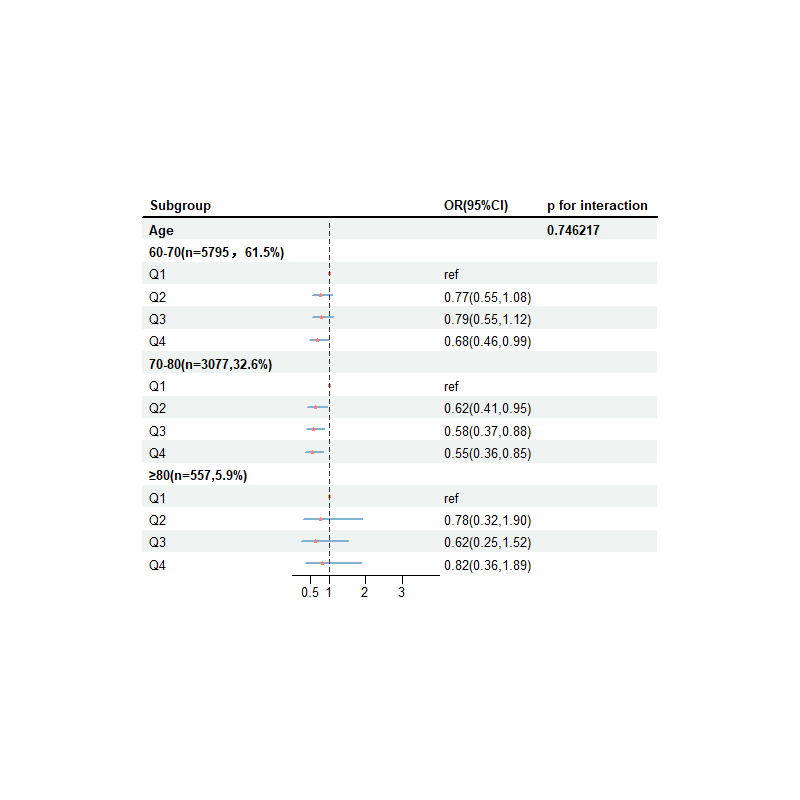

Supplement: Supplementary file 3 — Supplementary Material 3 [file 12877_2024_4959_MOESM3_ESM.tiff]

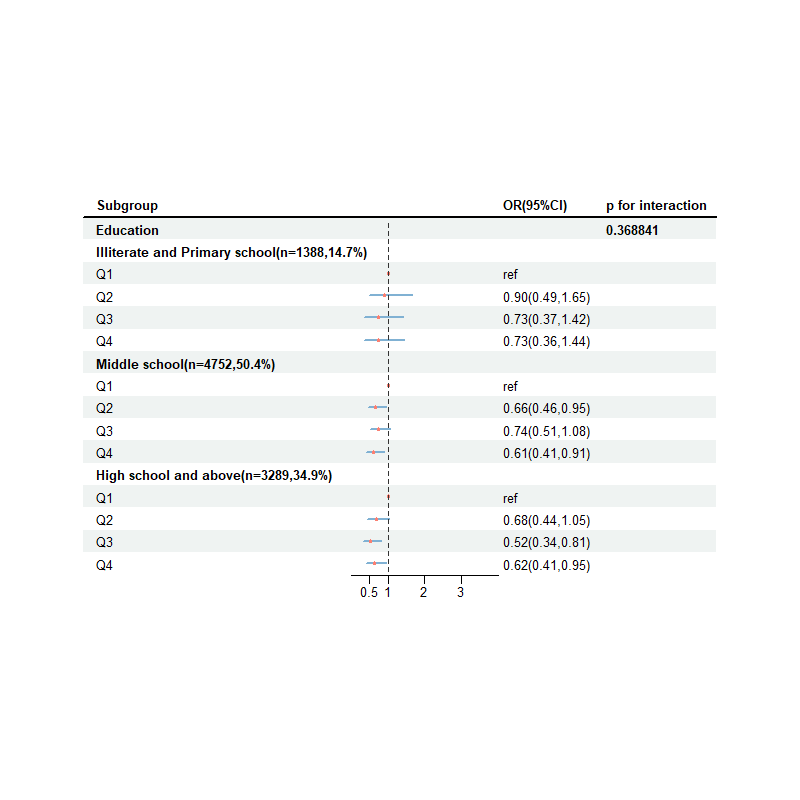

Supplement: Supplementary file 4 — Supplementary Material 4 [file 12877_2024_4959_MOESM4_ESM.tiff]

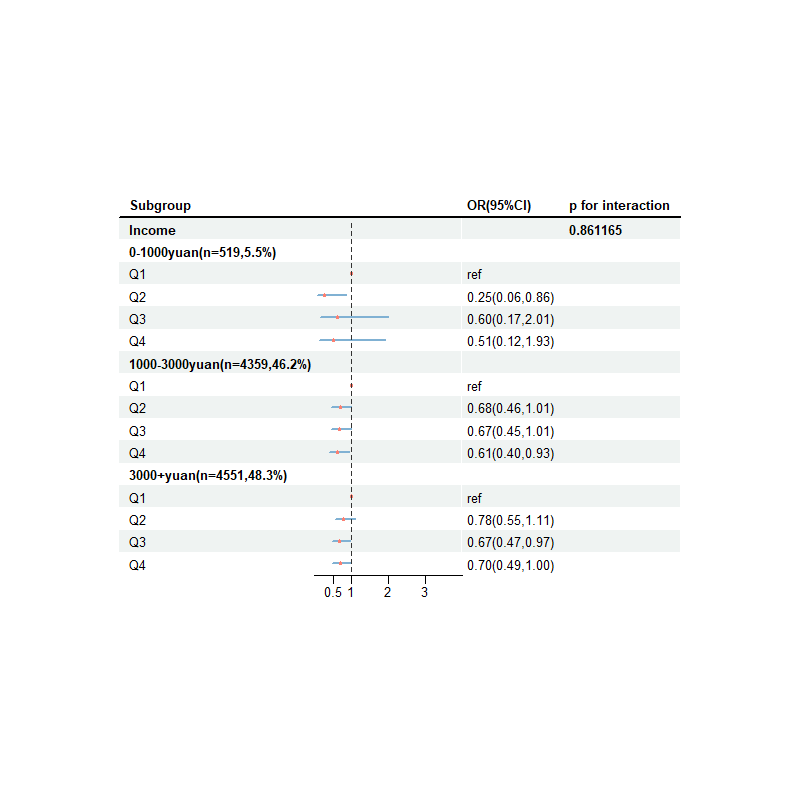

Supplement: Supplementary file 5 — Supplementary Material 5 [file 12877_2024_4959_MOESM5_ESM.tiff]

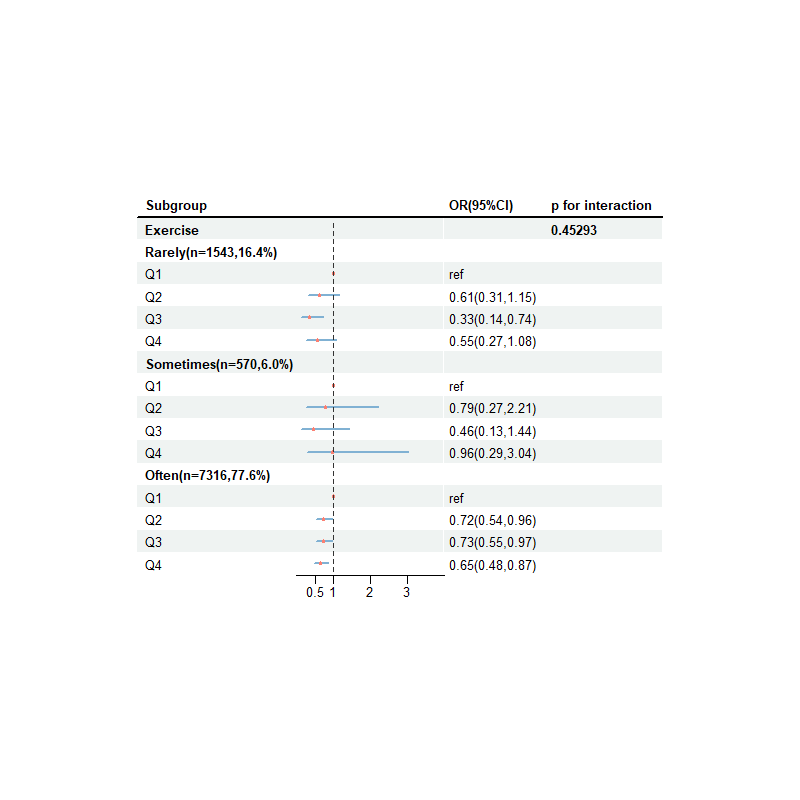

Supplement: Supplementary file 6 — Supplementary Material 6 [file 12877_2024_4959_MOESM6_ESM.tiff]

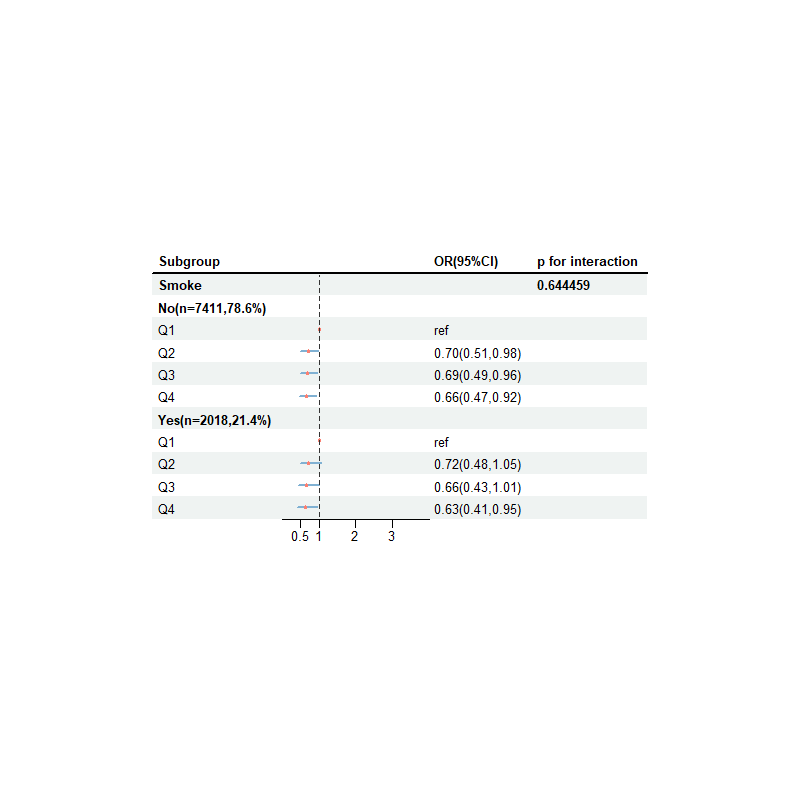

Supplement: Supplementary file 7 — Supplementary Material 7 [file 12877_2024_4959_MOESM7_ESM.tiff]

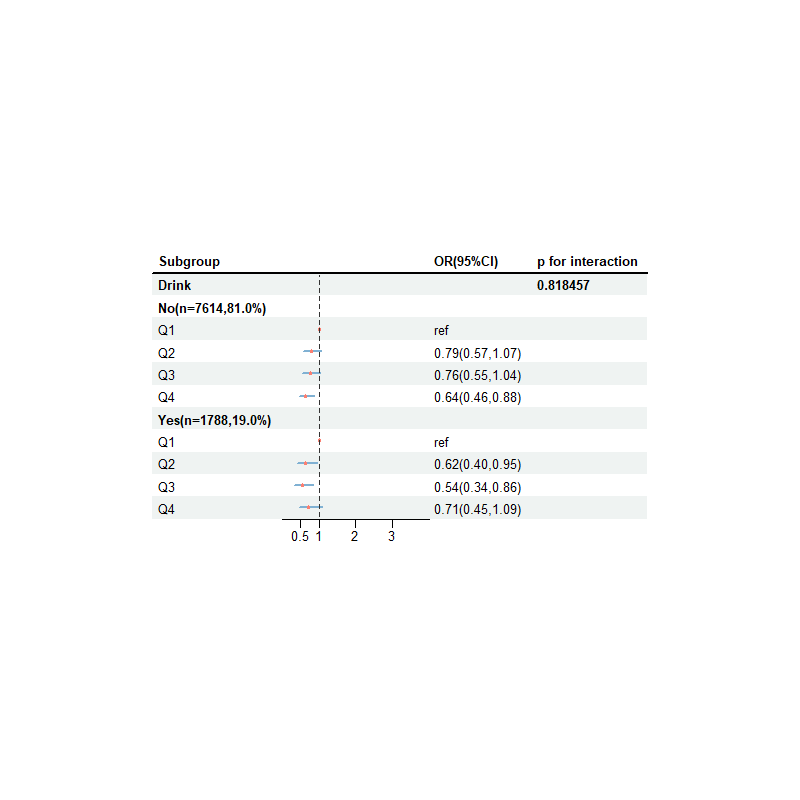

Supplement: Supplementary file 8 — Supplementary Material 8 [file 12877_2024_4959_MOESM8_ESM.tiff]

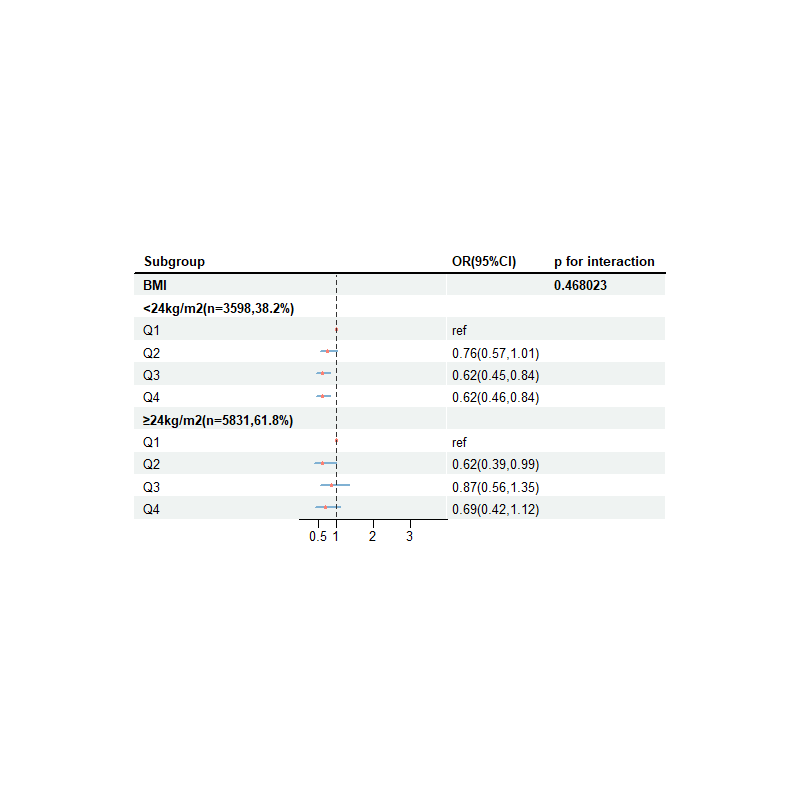

Supplement: Supplementary file 9 — Supplementary Material 9 [file 12877_2024_4959_MOESM9_ESM.tiff]
