# Supplementary material for: Lacto-ovo-vegetarian diet is inversely associated with the osteosarcopenia in older adults
Source: BMC Geriatr. 2024 Apr 11;24:332. doi: 10.1186/s12877-024-04959-6 (PMC11007993; doi:10.1186/s12877-024-04959-6)
Supplement: Supplementary file 10 — Supplementary Material 10 [file 12877_2024_4959_MOESM10_ESM.docx]

Supplementary Tabel 1. Factor loadings of the dietary patterns obtained by PCA.

|  | Aquatic products-meat | Lacto-ovo-vegetarian | Pork-sugar-oil |
| --- | --- | --- | --- |
| Rice | -0.103118382 | 0.013247091 | 0.447722638 |
| Wheat flour and products | -0.043257055 | 0.3008152 | 0.127445888 |
| Miscellaneous grains | 0.07414213 | 0.540689704 | -0.117373946 |
| Tubers | 0.157541295 | 0.330268163 | 0.051478603 |
| Pork | 0.148975096 | 0.098924019 | 0.546447673 |
| Beef and mutton | 0.573742796 | 0.172564265 | 0.283572963 |
| Poultry | 0.590066769 | 0.157833205 | 0.295868366 |
| Organ meats | 0.561714481 | -0.077360948 | 0.150956015 |
| Freshwater fish | 0.73866252 | 0.029312827 | -0.03413133 |
| Marine fish | 0.72191259 | 0.085302609 | -0.029728292 |
| Shrimp, crab and shellfish | 0.680890215 | 0.135318556 | -0.010049906 |
| Seaweeds | 0.455842348 | 0.21475512 | -0.141922357 |
| Fresh milk | 0.093143079 | 0.456644717 | -0.102001266 |
| Dairy products | 0.183651444 | 0.457775392 | -0.098151857 |
| Eggs | 0.055115543 | 0.467752488 | 0.026253924 |
| Soy products | 0.271900735 | 0.429703113 | -0.046596443 |
| Fruit | -0.101266414 | 0.560015907 | 0.080669241 |
| Vegetables | -0.251204414 | 0.349546147 | 0.285742666 |
| Nuts | 0.120986681 | 0.455738639 | 0.245489712 |
| Pastry and sweets | 0.064921023 | 0.216688097 | 0.483173825 |
| Fried foods | 0.155044727 | -0.10132044 | 0.472424216 |
| Sugar sweetened beverages | 0.001463177 | -0.081100631 | 0.353608773 |
| Proportion Var | 0.138 | 0.098 | 0.068 |
| Cumulative Var | 0.138 | 0.236 | 0.304 |
