# Supplementary material for: Lacto-ovo-vegetarian diet is inversely associated with the osteosarcopenia in older adults
Source: BMC Geriatr. 2024 Apr 11;24:332. doi: 10.1186/s12877-024-04959-6 (PMC11007993; doi:10.1186/s12877-024-04959-6)
Supplement: Supplementary file 11 — Supplementary Material 11 [file 12877_2024_4959_MOESM11_ESM.docx]

Supplementary Tabel 2. Baseline characteristics of the participants grouped by quartile groups of aquatic products-meat dietary pattern.

|  | Q1 | Q2 | Q3 | Q4 | p | p.trend |
| --- | --- | --- | --- | --- | --- | --- |
|  | N=2357 | N=2357 | N=2357 | N=2358 |  |  |
| Age | 68.0[65.0;73.0] | 68.0[65.0;72.0] | 67.0[65.0;71.0] | 68.0[65.0;72.0] | <0.001 | <0.001 |
| Gender: |  |  |  |  | <0.001 | <0.001 |
| Male | 717(30.4%) | 846(35.9%) | 893(37.9%) | 1044(44.3%) |  |  |
| Female | 1640(69.6%) | 1511(64.1%) | 1464(62.1%) | 1314(55.7%) |  |  |
| Ethnicity: |  |  |  |  | 0.066 | 0.654 |
| Han | 2226(94.4%) | 2236(94.9%) | 2253(95.6%) | 2214(93.9%) |  |  |
| Minority | 131(5.56%) | 121(5.13%) | 104(4.41%) | 144(6.11%) |  |  |
| Education: |  |  |  |  | <0.001 | <0.001 |
| Illiterate | 66(2.80%) | 51(2.16%) | 51(2.16%) | 54(2.29%) |  |  |
| Primary school | 408(17.3%) | 303(12.9%) | 215(9.12%) | 240(10.2%) |  |  |
| Middle school | 1174(49.8%) | 1205(51.1%) | 1220(51.8%) | 1153(48.9%) |  |  |
| High school and above | 709(30.1%) | 798(33.9%) | 871(37.0%) | 911(38.6%) |  |  |
| Income: |  |  |  |  | <0.001 | <0.001 |
| 0-1000 yuan | 192(8.15%) | 134(5.69%) | 110(4.67%) | 83(3.52%) |  |  |
| 1000-3000 yuan | 1173(49.8%) | 1123(47.6%) | 1009(42.8%) | 1054(44.7%) |  |  |
| 3000+ yuan | 992(42.1%) | 1100(46.7%) | 1238(52.5%) | 1221(51.8%) |  |  |
| Exercise: |  |  |  |  | 0.205 | 0.985 |
| Rarely | 411(17.4%) | 368(15.6%) | 363(15.4%) | 401(17.0%) |  |  |
| Sometimes | 123(5.22%) | 150(6.36%) | 152(6.45%) | 145(6.15%) |  |  |
| Often | 1823(77.3%) | 1839(78.0%) | 1842(78.2%) | 1812(76.8%) |  |  |
| Smoke: |  |  |  |  | <0.001 | <0.001 |
| Yes | 427(18.1%) | 499(21.2%) | 528(22.4%) | 564(23.9%) |  |  |
| No | 1930(81.9%) | 1858(78.8%) | 1829(77.6%) | 1794(76.1%) |  |  |
| Drink: |  |  |  |  | <0.001 | <0.001 |
| Yes | 320(13.6%) | 424(18.0%) | 478(20.3%) | 566(24.0%) |  |  |
| No | 2037(86.4%) | 1933(82.0%) | 1879(79.7%) | 1792(76.0%) |  |  |
| BMI group: |  |  |  |  | 0.817 | 0.967 |
| <18.5kg/m2 | 35(1.48%) | 39(1.65%) | 35(1.48%) | 40(1.70%) |  |  |
| <24kg/m2 | 880(37.3%) | 823(34.9%) | 882(37.4%) | 864(36.6%) |  |  |
| <28kg/m2 | 1040(44.1%) | 1061(45.0%) | 1021(43.3%) | 1034(43.9%) |  |  |
| ≥28kg/m2 | 402(17.1%) | 434(18.4%) | 419(17.8%) | 420(17.8%) |  |  |
