# Supplementary material for: Lacto-ovo-vegetarian diet is inversely associated with the osteosarcopenia in older adults
Source: BMC Geriatr. 2024 Apr 11;24:332. doi: 10.1186/s12877-024-04959-6 (PMC11007993; doi:10.1186/s12877-024-04959-6)
Supplement: Supplementary file 12 — Supplementary Material 12 [file 12877_2024_4959_MOESM12_ESM.docx]

Supplementary Tabel 3. Baseline characteristics of the participants grouped by quartile groups of lacto-ovo-vegetarian dietary pattern.

|  | Q1 | Q2 | Q3 | Q4 | p | p.trend |
| --- | --- | --- | --- | --- | --- | --- |
|  | N=2357 | N=2357 | N=2357 | N=2358 |  |  |
| Age | 67.0[64.0;71.0] | 68.0[65.0;71.0] | 68.0[65.0;72.0] | 69.0[65.0;73.0] | <0.001 | <0.001 |
| Gender: |  |  |  |  | 0.001 | <0.001 |
| Male | 947(40.2%) | 888(37.7%) | 835(35.4%) | 830(35.2%) |  |  |
| Female | 1410(59.8%) | 1469(62.3%) | 1522(64.6%) | 1528(64.8%) |  |  |
| Ethnicity: |  |  |  |  | 0.355 | 0.085 |
| Han | 2242(95.1%) | 2241(95.1%) | 2226(94.4%) | 2220(94.1%) |  |  |
| Minority | 115(4.88%) | 116(4.92%) | 131(5.56%) | 138(5.85%) |  |  |
| Education: |  |  |  |  | <0.001 | <0.001 |
| Illiterate | 61(2.59%) | 58(2.46%) | 55(2.33%) | 48(2.04%) |  |  |
| Primary school | 399(16.9%) | 308(13.1%) | 226(9.59%) | 233(9.88%) |  |  |
| Middle school | 1287(54.6%) | 1220(51.8%) | 1171(49.7%) | 1074(45.5%) |  |  |
| High school and above | 610(25.9%) | 771(32.7%) | 905(38.4%) | 1003(42.5%) |  |  |
| Income: |  |  |  |  | <0.001 | <0.001 |
| 0-1000 yuan | 191(8.10%) | 144(6.11%) | 101(4.29%) | 83(3.52%) |  |  |
| 1000-3000 yuan | 1218(51.7%) | 1110(47.1%) | 1029(43.7%) | 1002(42.5%) |  |  |
| 3000+ yuan | 948(40.2%) | 1103(46.8%) | 1227(52.1%) | 1273(54.0%) |  |  |
| Exercise: |  |  |  |  | <0.001 | <0.001 |
| Rarely | 523(22.2%) | 403(17.1%) | 309(13.1%) | 308(13.1%) |  |  |
| Sometimes | 178(7.55%) | 161(6.83%) | 137(5.81%) | 94(3.99%) |  |  |
| Often | 1656(70.3%) | 1793(76.1%) | 1911(81.1%) | 1956(83.0%) |  |  |
| Smoke: |  |  |  |  | <0.001 | <0.001 |
| Yes | 674(28.6%) | 509(21.6%) | 404(17.1%) | 431(18.3%) |  |  |
| No | 1683(71.4%) | 1848(78.4%) | 1953(82.9%) | 1927(81.7%) |  |  |
| Drink: |  |  |  |  | <0.001 | <0.001 |
| Yes | 564(23.9%) | 444(18.8%) | 391(16.6%) | 389(16.5%) |  |  |
| No | 1793(76.1%) | 1913(81.2%) | 1966(83.4%) | 1969(83.5%) |  |  |
| BMI group: |  |  |  |  | 0.009 | 0.001 |
| <18.5kg/m2 | 38(1.61%) | 45(1.91%) | 38(1.61%) | 28(1.19%) |  |  |
| <24kg/m2 | 818(34.7%) | 844(35.8%) | 848(36.0%) | 939(39.8%) |  |  |
| <28kg/m2 | 1047(44.4%) | 1037(44.0%) | 1065(45.2%) | 1007(42.7%) |  |  |
| ≥28kg/m2 | 454(19.3%) | 431(18.3%) | 406(17.2%) | 384(16.3%) |  |  |
