# Supplementary material for: Lacto-ovo-vegetarian diet is inversely associated with the osteosarcopenia in older adults
Source: BMC Geriatr. 2024 Apr 11;24:332. doi: 10.1186/s12877-024-04959-6 (PMC11007993; doi:10.1186/s12877-024-04959-6)
Supplement: Supplementary file 13 — Supplementary Material 13 [file 12877_2024_4959_MOESM13_ESM.docx]

Supplementary Tabel 4. Baseline characteristics of the participants grouped by quartile groups of pork-sugar-oil dietary pattern.

|  | Q1 | Q2 | Q3 | Q4 | p | p.trend |
| --- | --- | --- | --- | --- | --- | --- |
|  | N=2357 | N=2357 | N=2357 | N=2358 |  |  |
| Age | 68.0[65.0;72.0] | 68.0[65.0;72.0] | 68.0[65.0;72.0] | 67.0[65.0;72.0] | 0.047 | 0.011 |
| Gender: |  |  |  |  | <0.001 | <0.001 |
| Male | 759(32.2%) | 831(35.3%) | 898(38.1%) | 1012(42.9%) |  |  |
| Female | 1598(67.8%) | 1526(64.7%) | 1459(61.9%) | 1346(57.1%) |  |  |
| Ethnicity: |  |  |  |  | 0.561 | 0.563 |
| Han | 2233(94.7%) | 2228(94.5%) | 2223(94.3%) | 2245(95.2%) |  |  |
| Minority | 124(5.26%) | 129(5.47%) | 134(5.69%) | 113(4.79%) |  |  |
| Education: |  |  |  |  | 0.517 | 0.230 |
| Illiterate | 53(2.25%) | 59(2.50%) | 53(2.25%) | 57(2.42%) |  |  |
| Primary school | 295(12.5%) | 304(12.9%) | 310(13.2%) | 257(10.9%) |  |  |
| Middle school | 1202(51.0%) | 1166(49.5%) | 1185(50.3%) | 1199(50.8%) |  |  |
| High school and above | 807(34.2%) | 828(35.1%) | 809(34.3%) | 845(35.8%) |  |  |
| Income: |  |  |  |  | <0.001 | <0.001 |
| 0-1000 yuan | 122(5.18%) | 129(5.47%) | 164(6.96%) | 104(4.41%) |  |  |
| 1000-3000 yuan | 1196(50.7%) | 1122(47.6%) | 1055(44.8%) | 986(41.8%) |  |  |
| 3000+ yuan | 1039(44.1%) | 1106(46.9%) | 1138(48.3%) | 1268(53.8%) |  |  |
| Exercise: |  |  |  |  | 0.152 | 0.620 |
| Rarely | 401(17.0%) | 354(15.0%) | 400(17.0%) | 388(16.5%) |  |  |
| Sometimes | 120(5.09%) | 157(6.66%) | 147(6.24%) | 146(6.19%) |  |  |
| Often | 1836(77.9%) | 1846(78.3%) | 1810(76.8%) | 1824(77.4%) |  |  |
| Smoke: |  |  |  |  | <0.001 | <0.001 |
| Yes | 430(18.2%) | 457(19.4%) | 527(22.4%) | 604(25.6%) |  |  |
| No | 1927(81.8%) | 1900(80.6%) | 1830(77.6%) | 1754(74.4%) |  |  |
| Drink: |  |  |  |  | <0.001 | <0.001 |
| Yes | 347(14.7%) | 405(17.2%) | 475(20.2%) | 561(23.8%) |  |  |
| No | 2010(85.3%) | 1952(82.8%) | 1882(79.8%) | 1797(76.2%) |  |  |
| BMI group: |  |  |  |  | 0.569 | 0.047 |
| <18.5kg/m2 | 44(1.87%) | 42(1.78%) | 29(1.23%) | 34(1.44%) |  |  |
| <24kg/m2 | 883(37.5%) | 859(36.4%) | 863(36.6%) | 844(35.8%) |  |  |
| <28kg/m2 | 1030(43.7%) | 1036(44.0%) | 1054(44.7%) | 1036(43.9%) |  |  |
| ≥28kg/m2 | 400(17.0%) | 420(17.8%) | 411(17.4%) | 444(18.8%) |  |  |
